# A Distributed Wireless Camera System for the Management of Parking Spaces

**DOI:** 10.3390/s18010069

**Published:** 2017-12-28

**Authors:** Stanislav Vítek, Petr Melničuk

**Affiliations:** Faculty of Electrical Engineering, Czech Technical University in Prague, Technicka 2, 166 27 Prague, Czech Republic; melnicukpetr@gmail.com

**Keywords:** parking space, occupancy, smart city, internet of things, raspbery pi, low-power consumption, histogram of oriented gradients, support vector machine

## Abstract

The importance of detection of parking space availability is still growing, particularly in major cities. This paper deals with the design of a distributed wireless camera system for the management of parking spaces, which can determine occupancy of the parking space based on the information from multiple cameras. The proposed system uses small camera modules based on Raspberry Pi Zero and computationally efficient algorithm for the occupancy detection based on the histogram of oriented gradients (HOG) feature descriptor and support vector machine (SVM) classifier. We have included information about the orientation of the vehicle as a supporting feature, which has enabled us to achieve better accuracy. The described solution can deliver occupancy information at the rate of 10 parking spaces per second with more than 90% accuracy in a wide range of conditions. Reliability of the implemented algorithm is evaluated with three different test sets which altogether contain over 700,000 samples of parking spaces.

## 1. Introduction

Recent population growth in urban areas negatively affects vehicle traffic in city centers. In addition to the negative impact on the environment, finding a vacant space in parking lots during peak hours may be almost impossible. Shoup reports that, based on the sample of 22 studies performed in 11 cities (Detroit, Washington, New Haven, London, Freiburg, Jerusalem, Cambridge, Cape Town, New York, San Francisco, and Sydney) between 1927 and 2001, drivers spend on average eight minutes finding a vacant space [1]. Naturally, cruising time may vary, numerous studies performed in European cities (Netherland, Belgium) report average cruise time for on-street parking of 30 s and off-street parking 50 s [2].

Systems able to manage this problem can be categorized into counter-based, sensor-based, and image or video based. The first two categories have a couple of drawbacks: counter-based systems could help only with information about a total number of vacant spaces, a sensor-based system costs a lot because of the number of sensors required to cover the entire parking lot. However, the third category is usually considered as quite expensive and producing a significant amount of data, which are unable to transmit over the wireless network, the growth in low-cost, low-power sensing and communication technologies enables a wide range of physical objects and environments to be monitored in fine spatial and temporal detail. A network of dedicated low-power devices connected to the cloud then could be part of the Internet of Things (IoT) platform for smart cities [3].

The oldest video systems for automatic detection of vacant parking spaces were based on a simple calculation of the differences between consecutive frames [4]. Motion detection is not cost-effective because of the need to process large amounts of redundant data. The system requires continuous image acquisition, which does not provide room for any energy savings. Although systems using temporal characteristics are not efficient, their accuracy may be relatively high, as shown by recent works [5,6]. All the below-mentioned approaches do not consider the temporal link between the consecutive frames and solve each image individually.

The system described in [7] is computationally less demanding. The system determines decision about occupancy from the ratio of the number of edges calculated by Laplace’s operator in the defined areas. The authors report an accuracy of over 95% for one test sequence of a parking lot with 53 parking spaces. The robustness of the classification algorithm is, however, quite low when conditions are worse. Delibatov et al., [8] model a parking slot as a volume in the 3D space. This approach eliminates problems with overlaps. Parking spaces are semi-automatically marked and then modeled by the probability density that a pixel is part of the parking space. For detection, a rotationally invariant local binary pattern (LBP) descriptor with a support vector machine (SVM) classifier with a nonlinear core is used. The LBP-SVM classifier works on small blocks that can be classified as one of six classes: cars, land, trees, skies, buildings and the rest. The final result of the occupancy of the parking space is obtained by the weighted sum of the results of the block classification where the scales are given by the models of parking spaces.

A number of works are focused on testing of the algorithms’ robustness. De Almeida et al., [9] verify the usability of texture descriptors based on LBP and local phase quantization (LPQ) descriptors using the SVM classifier. The authors showed an accuracy of 99% when the classification algorithm was tested on the same view of the car park as it was trained. If a different view of the car park was selected for training, they reached an accuracy of around 89%. Algorithms were tested on the PKlot [10] database of almost 700,000 samples.

In addition to the descriptors already mentioned, there are works employing wavelet transform [11], scale-invariant feature transform (SIFT) [12], histogram of oriented gradients (HOG) [13,14], integral channel features [15], or Hue histogram descriptor [16]. Fusek et al., used AdaBoost based algorithm and also compared such approach with HOG descriptor [17].

From the point of view of reliability, it is worth mentioning the works focused on Convolutional Neural Networks (CNN). Amato et al., [18] describes the system using deep learning with accuracy better than 90%. CNN was trained to directly decide about the occupancy status of the individual parking spaces seen by the video cameras. The authors used the CNRPark dataset [19] consisting of roughly 12.000 labeled images and the CNRPark-EXT dataset including 150.000 labeled samples. Classification of one parking space takes around 300 ms Raspberry Pi model B.

The paper is organized as follows. Section 2 is dedicated to some aspects of camera systems design and Section 3 introduces proposed system. Section 4 discussed results including comparions with some already published papers and Section 5 describes real-world scenarios. Section 6 concludes the paper.

## 2. Design of Camera System

**Figure 1:**
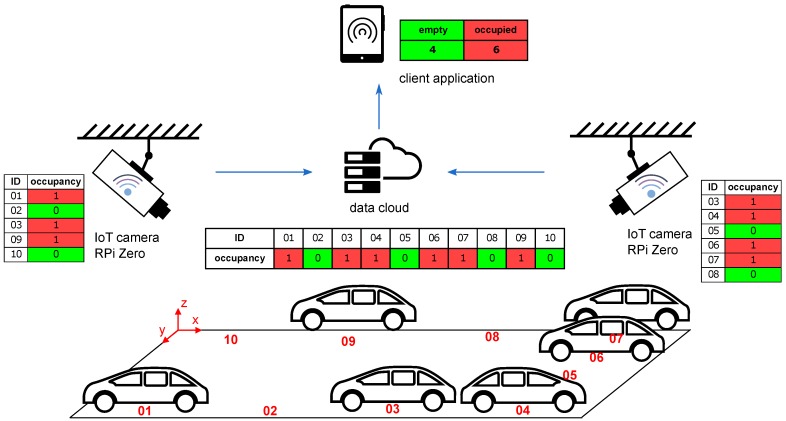
Multicamera parking slot management.

In this paper, we will focus on wireless camera systems. A typical arrangement of such a system can be seen in Figure 1. The first camera covers parking spaces 01, 02, 03, 09, and 10; the second camera covers parking spaces 03, 04, 05, 06, 07, and 08. Information on the occupancy of parking spaces is transferred to the server, which then evaluates the data and transmits it to the end user. In this case, one parking space (03) is visible from both cameras, so there should be an algorithm to solve this overlap. The server also has a database to keep all messages for further analysis.

It is sufficient to describe the occupancy of the single parking space with only 1 bit (0 = vacan/1 = occupied). The transfer of such information (including some error-correcting code) to the central server can be conveniently implemented via any of IoT wireless networks [20]. For example, the LPWAN (Low-Power Wide-Area Network) Sigfox platform allows users to transmit 96-bit messages 140 times a day, which approximately corresponds to an update interval of 10 min when sending regular messages. Since irregular intervals could better capture the peak situation, the time between messages can be adaptively modified according to the significance of changes in occupancy. Information about the occupancy of parking slots including navigation to the vacant space could be distributed via the Internet, for example using Vehicle-to-vehicle communication [21].

For wireless cameras, it is also necessary to solve their power supply. Cameras usually consume more energy than simpler sensors, and the design is therefore more complicated. The basic trick to reduce power consumption is the use of sleeping mode with a periodic wake-up. It is not necessary to update the parking capacity at a high sampling frequency, as slower changes are expected. When in sleeping mode, the power consumption of single unit can be minimized to the order of mW, thus significantly prolonging the battery life. Another way to optimize power consumption can be solar panels, which can achieve complete energy self-sufficiency of the camera [22].

**Figure 2:**
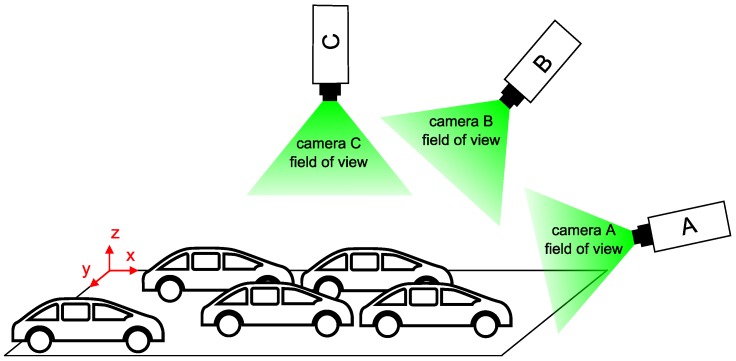
Possible camera locations.

An essential part of the design of camera systems is the consideration of the relative position of the cameras and the sensed objects. It is desirable to find the most efficient solution to maximize the coverage area with the minimum number of cameras. However, the design must take into account further consequences associated with placing the camera in a certain position [23]. The illustration image (Figure 2) indicates three different camera positions relative to the plane of the car park. The size of the area and the number of parking spaces to be covered by the camera depends on its location and its field of view. The large FOV usually associated with wide-angle lenses, intruding unwanted geometrical distortions. Camera A can easily cover all the parking spaces but at the cost of overlaps, which make occupancy harder to distinguish. Camera C can capture parking spaces without overlapping, but all of them come into view only when the camera is high above the ground or when using a large FOV lens. There may be overlaps in camera B but not as distinctive as in camera A. Option B seems to be the most advantageous as it is a compromise between the FOV and the overlaps. Moreover, the cameras can be placed on public lighting pillars or nearby buildings.

## 3. Implementation

Prototypes are based on Raspberry Pi Zero with camera module connected to the CSI-2 interface. Camera module uses CMOS sensors OmniVision OV5647 with a spatial resolution of 2592 × 1944 pixels and lens with focal length of 3.6 mm (FOV is 54 × 41 degrees). As a power supply we used two Li-Io batteries of 3.7 V (2 × 3.7 V × 1800 mAh) and DC-DC step-up converter 5 V/2 A.

The module is equipped with Wi-Fi adapter 802.11 b/g/n and LPWAN SigFox Node UART Modem working at 868 Mhz. While the Wi-Fi interface serves to configure the module, the SigFox modem serves to transmit information about the availability of parking spaces to the central server. Naturally, choice of this IoT communication platform limits the number of parking space that can be managed to approximately 90 (assuming that 6 bits will be used to identify camera module), which is enough for experimental purposes. All image processing tasks are done on Raspberry Pi, extracted information of vacant/occupied parking spaces is then transmitted through a central server to a client application.

### 3.1. Training Set and Descriptor

Training data are images displaying occupied and free parking spaces. Images were obtained with a camera mounted on a telescopic rod from a height of about 4 m. Overall, about 1000 images were collected in different daytimes and weather conditions. 1120 positive samples and 1378 negative were extracted from these data. After preliminary experiments we considered HOG descriptor as a most suitable and robust solution for our dataset. Test showed that HOG descriptor is highly invariant to light conditions. Extraction of feature vectors using the HOG descriptor requires input data of constant size, so the samples were resampled to the same size, aspect ratio 3:2.

**Figure 3:**
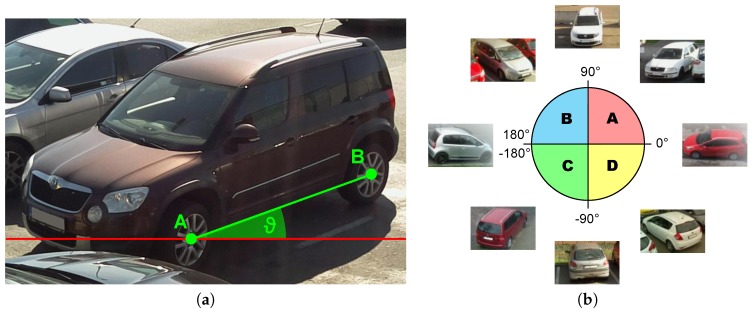
Orientation of the vehicle in the sample. (**a**) Example of the training sample with manually labeled wheels. (**b**) Demonstration of the similarity of differently rotated vehicles.

According to our experiments, the standard HOG feature descriptor is not able to adequately describe differently rotated vehicles and produces a high number of false positives. Our findings are supported by work of Fusek et al., [17], where authors report less than 44% accuracy of the HOG based detector. Thus, reasonably use the HOG descriptor requires describing the relative position of the car and the camera. For the purpose, we divided the positive samples into several categories according to the orientation of the car in the picture. The orientation is described by a single value corresponding to the angle between the wheels of the vehicle and the horizontal line. Figure 3a displays example of the training sample with manually labeled wheels (points *A* and *B*).

Although this model is very simple, it allows us to very easily distinguish the samples according to the orientation. The angle of the vehicle rotation can yield values from −180${}^{{}^{\circ}}$–180${}^{{}^{\circ}}$, but dimensionality can be reduced by adoption of the following simplification:

- it is possible to park a vehicle in two ways (for example −90${}^{{}^{\circ}}$ and 90${}^{{}^{\circ}}$, or −135${}^{{}^{\circ}}$ and 45${}^{{}^{\circ}}$), therefore, the samples corresponding to these angles can be merged;
- a typical vehicle is horizontally symmetric, so it is possible to group corresponding samples (for example 135${}^{{}^{\circ}}$ and 45${}^{{}^{\circ}}$).

**Figure 4:**
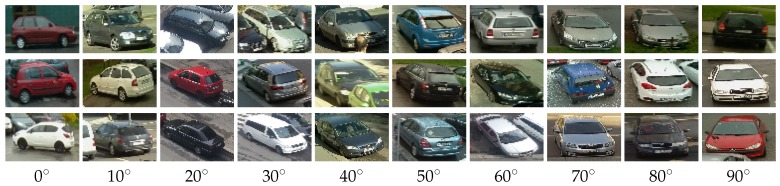
Groups of training samples.

Figure 3b demonstrates the similarity of differently rotated vehicles: quadrants A and B are horizontally symmetric, quadrants A and C represents opposite direction of parking and quadrants A and D show the opposite orientation of the cars in the parking space relative to the horizontal symmetry. Based on this consideration, we have merged positive samples into a single quadrant. After experiments, we resampled samples to the size of 72 × 48 pixels (i.e., feature vector has the length of 1440). This size of the sample was chosen as an optimal size according to the typical size of the car in the field of view of the camera. We divided samples into ten groups, representing vehicle rotations 0${}^{{}^{\circ}}$–90${}^{{}^{\circ}}$ with the step of 10${}^{{}^{\circ}}$. For examples see Figure 4.

### 3.2. Learning Algorithm

Since our target platform is Raspberry Pi Zero or possibly any other similar low-power platform, the main focus of the implementation is on efficiency and low complexity of used algorithms (i.e., lower usage of computer resources and thus lower power consumption). For this reason, we performed a comparison of popular learning algorithms performance: SVM with linear kernel, Logistic Regression (LR) and Random Forrest (RF). Tests were performed on our dataset described in Section 4.2, accuracy ACC (ratio between a number of correct classification and a total number of samples), area under ROC (Receiver operating characteristic) and also the average time needed to the classification of one parking space have been investigated. Results are summarized in the Table 1. The best learning algorithm regarding speed is LR, but this algorithm exhibits bad performance with our dataset. As the best trade-off between computational difficulty and accuracy, we selected SVM. Due to the relatively large size of the feature vector and a small number of samples, the training set data is linearly separable. Thus, the linear kernel is a natural choice.

### 3.3. Classification of the Parking Space

**Figure 5:**
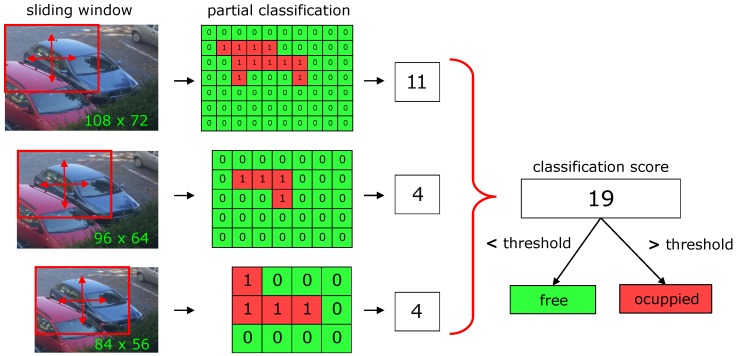
Classification of the single parking space.

Unlike the training phase, it is not possible to specify the position of the vehicle during classification, since its exact position and size are not known. Thus, the algorithm works with a previously known approximate position of a parking space, which is naturally larger than the vehicle itself. The classification is then carried out using a sliding window in variously resampled copies of the section of the parking area (see Figure 5). From each copy, a sliding window of size 72 × 48 produces several segments for which the HOG feature vectors are calculated. SVM classification model then predicts the class. If the model classifies the given feature vector as a vehicle, the prediction is equal to one. Otherwise, the prediction value is zero. Summing up the results for all segments produced by sliding window, the algorithm calculates a classification score that is thresholded to obtain the final classification result. The value of the threshold is determined from the statistical analysis, for details see Section 4. The classification of a single parking space is relatively computationally inexpensive; with Raspberry Pi Zero it takes about 100 ms. Thus, the system can classify about ten parking spaces per second.

If the system uses more cameras with an overlapping field of view, the parking space in the overlay is classified by all cameras. The decisive classification score is the value with the greatest difference from the chosen threshold.

### 3.4. Power Consumption

The module works with the voltage of 5V. We measured the current consumption of the module during typical actions: idle, dense scanning (classification of single parking space), Wi-Fi connected and SigFox data transmission. For details about current consumption see Table 2. Note, that the Raspberry Pi Camera Modules require 250 mA to an operation, and the SigFox module has current consumption 65 mA during transmit and 15 mA during receive operation, typical sleep mode current is 2 μA. The timing of the actions is determined by the frequency of data transmissions and a number of parking spaces. Assuming that the module is working during the daytime, with the limitation of the SigFox network it is possible to send one message per five minutes. A Wi-Fi interface is switched on during normal operation, and it may be switched on with a command sent over the SigFox network. Peak current occurs when the camera is in action. This is typically 10 s during the above-mentioned five minutes interval (to classify up to 100 parking spaces). The average power consumption of the single module is about 1.5 W during daytime (6 am to 8 pm during the summer).

## 4. Validation of the Results

For the purpose of testing the entire system, validation of the results was carried out on the databases PKLot and two smaller databases collected by the authors of the article—PKSlots described in Section 4.2 and FELSlot described in Section 4.3.

### 4.1. PKLot

**Figure 6:**
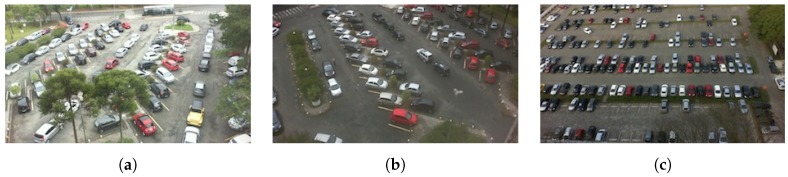
Parking lots used in PKLot database (**a**) Federal University of Parana; (**a**) Federal University of Parana, captured from 4th floor of the building; (**b**) Federal University of Parana, captured from 5th floor of the building; (**c**) Pontifical Catholic University of Parana, captured from 10th floor of the building.

PKLot database was created from 12,417 images of size 1280 × 720 pixels captured with low-cost HD camera on two different parking lots in sunny, cloudy and rainy days. The first parking lot was captured in two different view angles, so there are three different datasets (see Figure 6, images taken from [10]). The whole dataset contains 695,899 samples of parking spaces, but since some of the samples have missing info about occupancy, we used only 667,076 of them (348,125 negative and 318,951 positive samples).

**Figure 7:**
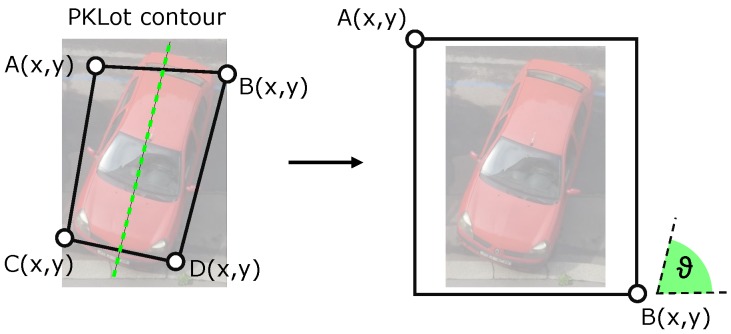
Transformation of PKLot.

The format of data definitions used in the PKLot database is different from the data format we have designed for our system. The PKLot defines the parking space using a contour close to the assumed position of the car and automatically calculates the parameters of the rotated rectangle from contour coordinates, see Figure 7. However, the parameters of the rectangle were not satisfactory for the separation of the parking spaces. The main problem was that the angle parameter often does not correspond to the actual orientation of the parking space. We solved this problem by using the original contours, from which we constructed a straight line (green dashed line in Figure 7) whose angle we considered to be the orientation of the place.

**Figure 8:**
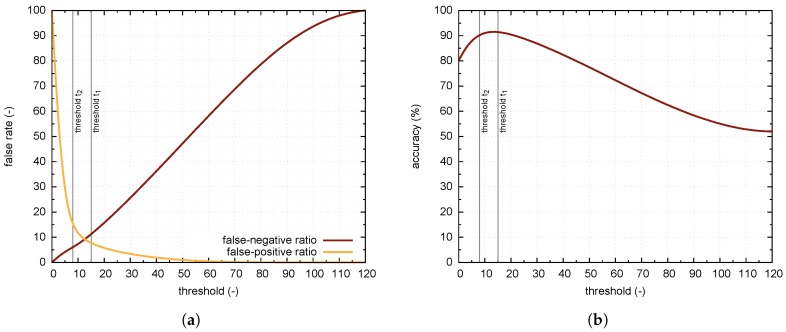
Statistical evaluation of algorithm performance (**a**) Threshold influence to the classification false rate; (**b**) Threshold influence to accuracy (ACC) of the classification.

Figure 8a shows the dependence of the false-rate of classification on the threshold setting, with the distinction whether a vacant or occupied place is recognized. Figure 8b displays accuracy ACC, the maximum value of ACC is 91.8% for the threshold equal to 15. Maximum accuracy, however, may not be the most important criterion when setting the threshold for a classification score. Efforts can be made to reduce false negatives (FN, i.e., algorithm did not recognize occupied place) even at the cost of precision, as lower FN is suited when submitting information to the user. For example, let have the threshold set to value 8, the accuracy of ACC reduces to 90.7%, but the number of situations where the system denotes the occupied parking space as vacant decreases to 6%. Table 3 shows confusion matrix for PKLot dataset and two values of the threshold. For the sake of completeness, note, that true positive (TP) means that algorithm correctly recognized car occupying parking space, true negative (TN) means that algorithm correctly recognized vacant space and false positive (FP) means that algorithm did not recognize vacant place. Since authors of the PKLot database labeled samples also in terms of weather, it is possible to study the impact of weather conditions to algorithm performance. Table 4 shows the confusion matrix corresponding to PKLot samples obtained in cloudy, rainy or sunny weather.

### 4.2. PKSlots

**Figure 9:**
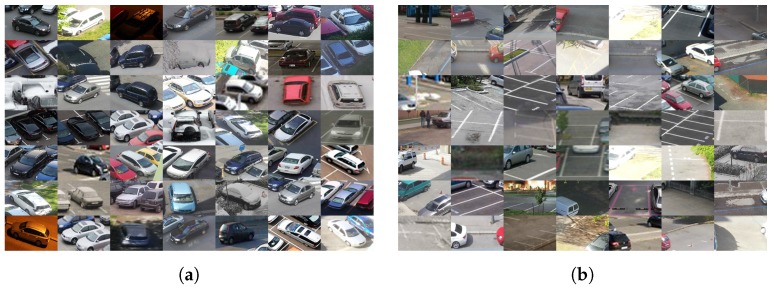
PKSlots dataset (**a**) Positive samples; (**b**) Negative samples.

PKSlots is a custom dataset collected by the authors of this paper. The dataset consists of 774 samples of parking slots that were obtained from a total of 142 very different shots (different angles, different weather conditions, etc.) taken by the use of different cameras under different conditions. See Figure 9 for examples of both positive and negative samples.

Using ROC analyses we determined the optimal threshold (t=8). Table 5 displays a confusion matrix for this value of threshold. Of the 774 samples, only 53 (25 cars and 28 vacancies) were incorrectly classified, and accuracy reached a value of 93.2%. Due to the significant variability of the images, this is a very satisfactory result, and it is also good that the FN value is lower than FP.

### 4.3. FELSlot

**Figure 10:**
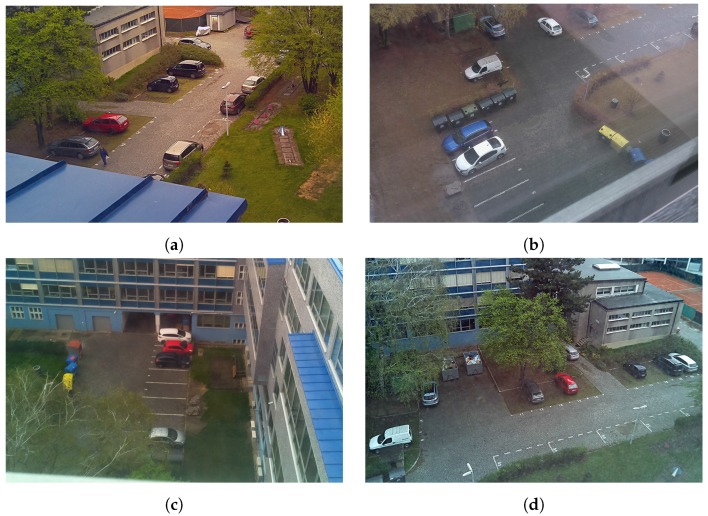
FELSlot dataset (**a**) Position A1–14 parking slots, 1615 images; (**b**) Position A1–14 parking slots, 695 images; (**c**) Position B1–8 parking slots, 1635 images; (**d**) Position B2–16 parking slots, 1604 images.

Testing dataset FELSlot is another dataset collected by authors of this paper. It consists of 5.549 images of the parking slot at the Faculty of Electrical Engineering of Czech Technical University in Prague. Cameras (IoT RaspberyPi modules described in this paper) were placed in four different positions, 71.084 samples were obtained in total during two consecutive days. For examples see Figure 10.

Table 6 shows confusion matrix for the threshold t=15. Accuraccy is then ACC=92.2%.

### 4.4. Comparison with Other Algorithms

To evaluate the performance of our method, we tried to compare the results we achieved with other known algorithms. Because the authors of papers typically present the results in different forms, the following text is a simple comparison without a deeper analysis. Baroffio et al., tested algorithm based on Hue histogram and linear SVM [16] and published accuracy reached on PKLot database (UFPR04—96%, UFPR05—93%, PUCPR—87%). Amato et al., [18,19] refer accuracy of their CNN based method up to 99%, but their results strongly depend on the choice of the learning dataset. Almeida et al., [9], authors of PKLot database, used SVMs trained on histograms of textural features. Their paper refers to following accuracy: UFPR04—99%, UFPR05—84%, PUCPR—84%. Fusek et al., [17] refer accuracy of HOG based detector 44% and accuracy of the detector based on AdaBoost 94%. Our method, trained on our custom dataset, reached an accuracy of 96% on UFPR04, 83% on UFPR05 and 94% on PUCPR.

## 5. Working Scenarios

The system described in this paper is able to work in two basic modes: (a) mode with predefined parking places and (b) mode with parking places determined automatically.

### 5.1. Predefined Parking Spaces

**Figure 11:**
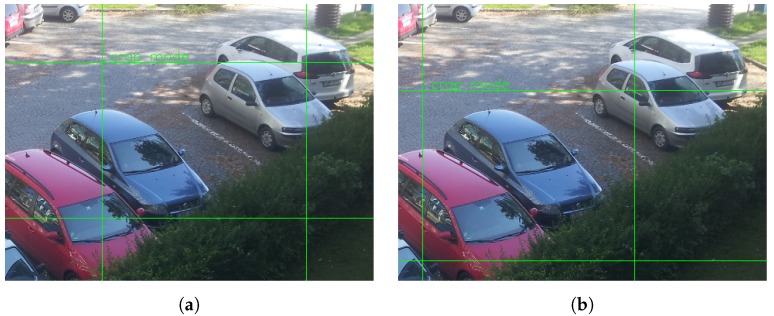
Manual definition of two neighboring parking spaces. (**a**) Example of vacant parking space. (**b**) Example of occupied parking space.

In this mode, an operator has to select and label expected parking spaces. For this purpose, the operator can connect the module using Wi-Fi interface and use GUI (graphical user interface) to define parking places—see Figure 11. In case of cameras with overlapping fields of view, an operator has to label parking spaces in overlapped areas; the occupancy is then evaluated as described in Section 3.3. Evaluation of parking spaces in overlapped areas is done in the central server. In this scenario, users receive information about the occupancy of a particular parking space.

### 5.2. Determination of Parking Space Position

The previous working scenario, described in Section 5.1, depends on the input of the operator. In the more challenging scenarios, like on-street parking, a position of parking spaces may change in time. Thus, we propose to use a simple procedure to determine and update a position of parking places. For this purpose, the procedure employs the single-pass version of classification algorithm described in Section 3.3, for details see Algorithm 1.

**Algorithm** **1**: Algorithm of determination of the vehicles position
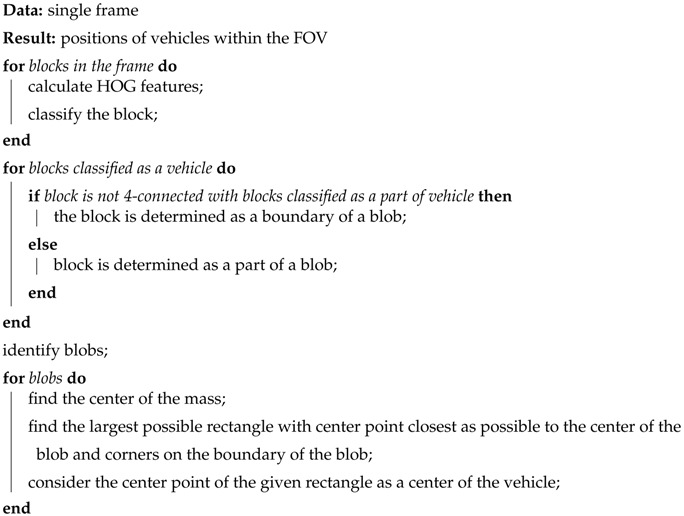


**Figure 12:**
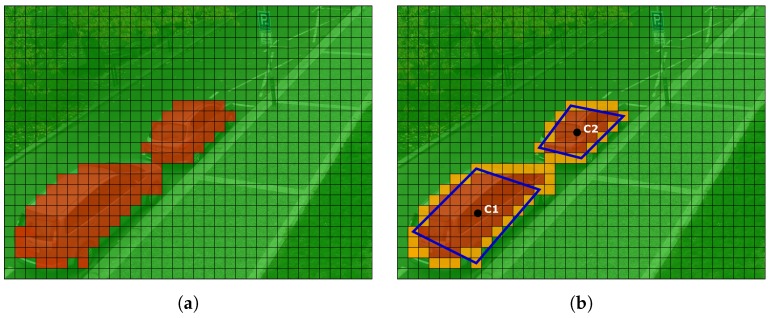
Determination of parking spaces position. (**a**) Blocks classified as a vehicle. (**b**) Boundaries of the blobs and detemination of the vehicle position.

Figure 12a display an example, how the frame is processed. Set of blocks classified as a vehicle take a form of a blob (red color). Blocks belonging to the blob with less than 4-connectivity with other blocks in the blob are considered as a blob boundary (yellow color). Determination of the blob boundaries allows us to divide the blob into a few smaller ones that fit the position of the vehicles. The algorithm then estimates centers of mass of a new blob (or blobs, if there are more than one car—see Figure 12b). Rectangles estimates around the blobs then define a position of parking spaces.

This procedure can be run for a couple of days after installation of the module to train positions of the available parking spaces and on the regular basis to update them. When about ten cars are present in the FOV, the procedure takes about 90 s; current consumption is the same as for classification.

## 6. Conclusions

A fast and reliable algorithm designed to run on low-power consumption embedded systems is used to classify images of parking space to recognize if they are occupied or vacant. The extracted information can be used in any application aiming to help drivers to find free parking space. In order to increase the accuracy of the detector based on HOG descriptors and SVM, we have provided a descriptor with additional information about rotation of the vehicle. We also employed dense scanning in three different scales to improve detector performance.

Performance of our algorithm was determined on the publicly available dataset for parking occupancy detection PKLot. We also collected two new datasets based on the different approach (more views and weather conditions) and then evaluated them. Average time to classify one parking space is about 100 ms on Raspberry Pi Zero. For the real-world applications, we prepared two different scenarios that are able to manage off-street as well as on-street parking, with a self learning procedure. Clearly, there is room for further improvement, for example, the design of predicting algorithms. Tests in the real environment showed that the system works well for a wide range of light conditions. We would also like to solve possible security issues—our plan is to implement our system with the use of IBM Bluemix, which is employing secured MTTQ (Message Queue Telemetry Transport) protocol.

## Figures and Tables

**Table 1:** Comparison of learning algorithms.

	ACC	ROC	Time
RF	0.910	0.946	110 ms
SVM	0.931	0.955	100 ms
LR	0.638	0.782	30 ms

**Table 2:** Current consumption of the module.

Activity	Current
idle	80 mA
dense scanning	830 mA
Wi-Fi connected	250 mA
data transmission	145 mA

**Table 3:** PKLot dataset: confusion matrix for two different thresholds.

$t_{1}=15$				$t_{2}=8$			
**TP**	285,542	**FN**	33,409	**TP**	299,812	**FN**	19,139
	89.5%		10.5%		94.0%		6.0%
**FP**	21,562	**TN**	326,563	**FP**	42,566	**TN**	305,559
	6.2%		93.8%		12.2%		87,7%
ACC = 91.8%				ACC = 90.7%			

**Table 4:** PKLot dataset: confusion matrix for different weather conditions, threshold $t_{2}=15$.

Cloudy				Rainy				Sunny			
**TP**	77,663	**FN**	8426	**TP**	55,803	**FN**	3177	**TP**	152,076	**FN**	21,806
	90.2%		9.8%		94.6%		5.4%		87.5%		12.5%
**FP**	9358	**TN**	130,677	**FP**	2110	**TN**	31,478	**FP**	10,094	**TN**	164,408
	6.7%		93.3%		6.3%		93,7%		5.8%		94.2%
ACC = 92.1%				ACC = 94.3%				ACC = 90.8%			

**Table 5:** Confusion matrix for PKSlots dataset, *t* = 8.

**TP**	423	**FN**	25
	94.4%		5.6%
**FP**	28	**PN**	298
	8.6%		91.4%
ACC = 93.2%			

**Table 6:** Confusion matrix for FELSlot dataset, *t* = 15.

**TP**	27.639	**FN**	4784
	85.2%		14.8%
**FP**	773	**PN**	37,888
	2.0%		98.0%
